# A Molecular Mechanism for Bacterial Susceptibility to Zinc

**DOI:** 10.1371/journal.ppat.1002357

**Published:** 2011-11-03

**Authors:** Christopher A. McDevitt, Abiodun D. Ogunniyi, Eugene Valkov, Michael C. Lawrence, Bostjan Kobe, Alastair G. McEwan, James C. Paton

**Affiliations:** 1 Research Centre for Infectious Diseases, School of Molecular and Biomedical Science, University of Adelaide, Adelaide, South Australia, Australia; 2 School of Chemistry and Molecular Biosciences, Australian Infectious Diseases Research Centre and Institute for Molecular Bioscience, University of Queensland, Brisbane, Australia; 3 The Walter and Eliza Hall Institute of Medical Research, Parkville, Victoria, Australia; University of Illinois, United States of America

## Abstract

Transition row metal ions are both essential and toxic to microorganisms. Zinc in excess has significant toxicity to bacteria, and host release of Zn(II) at mucosal surfaces is an important innate defence mechanism. However, the molecular mechanisms by which Zn(II) affords protection have not been defined. We show that in *Streptococcus pneumoniae* extracellular Zn(II) inhibits the acquisition of the essential metal Mn(II) by competing for binding to the solute binding protein PsaA. We show that, although Mn(II) is the high-affinity substrate for PsaA, Zn(II) can still bind, albeit with a difference in affinity of nearly two orders of magnitude. Despite the difference in metal ion affinities, high-resolution structures of PsaA in complex with Mn(II) or Zn(II) showed almost no difference. However, Zn(II)-PsaA is significantly more thermally stable than Mn(II)-PsaA, suggesting that Zn(II) binding may be irreversible. *In vitro* growth analyses show that extracellular Zn(II) is able to inhibit Mn(II) intracellular accumulation with little effect on intracellular Zn(II). The phenotype of *S. pneumoniae* grown at high Zn(II):Mn(II) ratios, *i.e.* induced Mn(II) starvation, closely mimicked a Δ*psaA* mutant, which is unable to accumulate Mn(II). *S. pneumoniae* infection *in vivo* elicits massive elevation of the Zn(II):Mn(II) ratio and, *in vitro*, these Zn(II):Mn(II) ratios inhibited growth due to Mn(II) starvation, resulting in heightened sensitivity to oxidative stress and polymorphonuclear leucocyte killing. These results demonstrate that microbial susceptibility to Zn(II) toxicity is mediated by extracellular cation competition and that this can be harnessed by the innate immune response.

## Author Summary

Dietary zinc deficiency is a global health problem affecting almost two billion people. Infectious diseases associated with zinc deficiency include respiratory infections caused by bacteria, and notably, *Streptococcus pneumoniae,* which is responsible for more than 1 million deaths annually. The association between zinc and immunity is well known, but the mechanism by which zinc provides protection against infectious diseases has remained a mystery. Previously, we found that manganese was essential for *S. pneumoniae* growth and its ability to cause disease. Intriguingly, we also observed that zinc could bind to the manganese transport protein. Therefore, we sought to determine if zinc could inhibit manganese transport, and to observe what the effects would be on *S. pneumoniae*. We found that zinc prevented manganese uptake. This slowed bacterial growth and rendered it hypersensitive to immune cell killing. We also observed that, during *S. pneumoniae* infection in mice, zinc released by the host increased to concentrations that could compete for manganese uptake. Our study provides direct evidence for how zinc is toxic to bacteria by preventing manganese uptake. Furthermore, we show how this could be harnessed by the immune system, thereby providing a scientific basis for the protective effect of zinc against infectious diseases.

## Introduction

*S. pneumoniae* is the world's foremost bacterial pathogen and a leading cause of death in young children in developing countries [1], [2], [3]. One of the major factors associated with the incidence and severity of *S. pneumoniae* infections in these children is dietary zinc deficiency (a significant ongoing problem in developing countries [4], [5]). Zinc, which occurs as the divalent cation Zn(II), is the second most abundant transition metal in humans and has crucial roles in many facets of the immune system [6], [7]. The physiological concentration ranges of Zn(II) range from a few µM to over 100 µM and it has been suggested that Zn(II) interacts with up to 10% of all human proteins [8], [9], [10]. Zn(II) concentrations are elevated in response to inflammation and bacterial infection as a consequence of Zn(II) release from damaged or apoptotic cells, and from sequestering proteins such as metallothionein. Despite the requirement of Zn(II) for optimal immune function, diseases in developing countries associated with Zn(II)-poor status are predominantly acute respiratory infections, otitis media and diarrhea [7]. In recent years clinical trials of Zn supplementation have been undertaken in developing countries and meta-analyses of multiple trials [11], [12] have shown a clear association between Zn(II) supplementation and a reduction in the incidence and severity of pneumonia and diarrhea. However, to date no clear mechanism for the protective effect of Zn(II) has been identified.

Zn(II) is also an essential micronutrient for bacteria, although it has significant toxicity at high concentrations [13], [14], [15], [16], [17]. However, the molecular basis of zinc toxicity remains poorly defined. Studies from our laboratory have previously demonstrated that the pneumococcal surface antigen A (PsaA) from *S. pneumoniae* has a clear interaction with Zn(II) [18] despite not being involved in its uptake, which occurs via a distinct Zn(II) ATP-binding cassette (ABC) permease [19], [20]. PsaA is the solute-binding protein (SBP) of a manganese-specific ABC permease encoded by the *psaBCA* locus. Mn(II) is an essential trace element for both prokaryotes and eukaryotes where it has roles in many cellular processes [21]. In *S. pneumoniae* Mn(II) regulates a diverse array of genes and has been shown to have roles in competence and in managing oxidative stress [22], [23]. Notably, the importance of Mn(II) for oxidative stress management, beyond its role in manganese superoxide dismutase, is an area of growing research. Recently, Mn(II) was shown to be able to substitute for ferrous iron in a nonredox metabolic enzyme to protect carbon metabolism under conditions of oxidative stress [24]. Intriguingly, both Mn(II) and Zn(II) are acquired by SBPs belonging to the Cluster A-1 family [25] of prokaryotic transporters [19]. The tertiary structure of the respective SBPs are very similar, although key differences are present at the metal binding sites [26]. Mn(II) acquisition, mediated by PsaA, is important for *S. pneumoniae* growth, proliferation and virulence [27]. In *S. pneumoniae,* loss of *psaA* results in a massive reduction of virulence in systemic, respiratory tract and otitis media murine models of infection [27], [28], [29]. The importance of Mn(II) for the virulence of bacteria has also been observed in a number of other pathogens including *Bacillus anthracis, S. pyogenes,* and *Staphylococcus aureus* [30], [31], [32]. Therefore, as the *S. pneumoniae* Mn(II) ABC permease PsaBCA is essential for growth *in vivo*, we hypothesized that Zn(II) could compete for Mn(II) binding, and thereby mediate toxicity by impairing Mn(II) acquisition.

In this paper, we present the biophysical characterization of PsaA and show how Zn(II) competition for Mn(II) transport *in vitro* impairs growth and renders *S. pneumoniae* hypersensitive to oxidative and polymorphonuclear leukocyte killing. We then illustrate the significance of these findings by showing how *in vivo* host Zn(II) concentrations change in response to *S. pneumoniae* infection. Taken together, this study presents a new paradigm for the molecular basis of bacterial susceptibility to Zn(II) toxicity, due to its extracellular competition for an essential metal ion micronutrient transporter, and its exploitation *in vivo* by the host immune response.

## Results

### PsaA is a high affinity Mn solute binding protein

**Figure 1:**
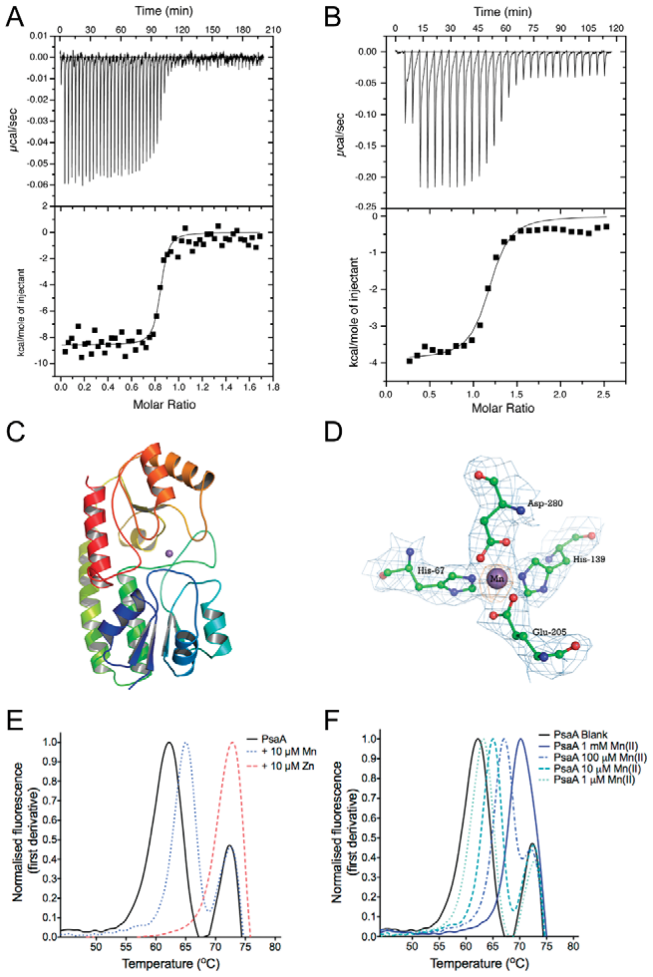
Biophysical characterization of purified PsaA. (A) Representative ITC measurements for titration of 4.5 µM PsaA with 40 µM Mn(II). (B) Titration of 20 µM PsaA with 250 µM Zn(II). For each experiment the rates of heat release are shown above the corresponding plots of integrated heat. Both of the curves were fitted to a single site (*n* = 1) model and the K*_D_* calculated from replicate experiments (± SEM). (C) The overall fold of PsaA with the metal ion shown in purple between the two domains. (D) The metal binding site. The 2F_O_-F_c_ electron density map (contoured at the 1.0σ level) is shown in blue for the coordinating residues and the metal ion. The Mn(II) is shown as a purple sphere and the residues are in ball-and-stick representation (carbon atoms in green, oxygen in red and nitrogen in blue). Also shown in orange is the anomalous difference Fourier map contoured at the 5.0 σ level, computed using the Bijvoet differences collected at the manganese K-edge peak wavelength. (E) Thermal stability of PsaA. The sets of curves show the thermal transition of 10 µM PsaA incubated with 10 µM Mn(II) or 10 µM Zn(II). The curves are representative of three independent experiments (*n* = 3). (F) Thermal stability of PsaA with increasing Mn(II) concentrations as indicated.

We first determined the binding constants (K*_A_*) of Mn(II) and Zn(II) with PsaA using isothermal titration calorimetry (ITC). Representative binding isotherms of *apo*-PsaA are shown in Fig. 1A and B (for further details see Text S1). The derived affinity constants (^1^/K*_A_* = K*_D_*) calculated for 1∶1 complexes of PsaA with Mn(II) or Zn(II) were 3.3±1.0 nM and 231±1.9 nM, respectively. The ITC data indicated that Zn(II) had an affinity nearly two orders of magnitude lower than Mn(II), consistent with the role of PsaBCA in Mn(II) acquisition under physiological conditions. Nevertheless, the data suggested Zn(II) could compete for PsaA binding at high concentrations.

### Mn(II) and Zn(II) bind to PsaA with no apparent structural difference to the protein

The crystal structure of PsaA had previously been determined with Zn(II) tetrahedrally coordinated by the four metal-binding residues (His67, His139, Glu205 and Asp280) [18] but no structure for the Mn(II)-loaded protein has been reported. We crystallized Mn(II)-loaded PsaA and determined its structure at 2.7 Å resolution (PDB ID: 3ZTT). Well-defined and continuous electron density was observed for the active site residues that tetrahedrally coordinated the metal ion (Fig. 1C and D), which was identified as Mn(II) (see Text S2 for further details). The tetrahedral coordination of Mn(II) is analogous to Zn(II) coordination in PsaA (Fig. S1) and the structures of other Cluster A-1 SBPs (*Treponema pallidum* TroA [33], *Synechocystis* 6803 MntC [34], *Synechocystis* 6803 ZnuA [35] and *E. coli* ZnuA [26]). Overall, our structural analysis indicates that PsaA is capable of binding Mn(II) and Zn(II) with only very minor accompanying structural changes in the final loaded state (Fig. S1C and D).

Metal ion discrimination within the Cluster A-1 SBPs is proposed to occur through subtle differences in the binding sites, Zn(II)-binding being favoured by the presence of three N ligands (from His residues), and Mn(II)-binding being favoured by two N (His) and two O (Asp or Glu) ligands [26]. We determined, by ITC, that Zn(II) binding had a high enthalpy (ΔH =  −18.2±0.4 kcal.mol^−1^) relative to Mn(II) (ΔH =  −7.5±1.1 kcal.mol^−1^), as would be predicted by the Irving-Williams series for the stability of metal ion complexes [36]. However, the preference of PsaA for Mn(II) is the result of the highly unfavourable entropic contribution of Zn(II) binding (−TΔS = 9.5±0.4 kcal.mol^−1^) relative to Mn(II) (−TΔS =  −4.0±0.9 kcal.mol^−1^). Thus, the binding free energies (ΔG) of the PsaA-Mn(II) and PsaA-Zn(II) complexes (−11.5±0.2 kcal.mol^−1^ and −8.9±0.2 kcal.mol^−1^, respectively) are consistent with both the preference for Mn(II) under physiological conditions and the observed difference in affinity of the protein (1 order of magnitude  = 1.41 kcal.mol^−1^). Mn(II) preference for PsaA is, therefore, due to differences in the entropic contributions.

### Metal ion effects on PsaA thermal stability

We employed a thermal stability assay (TSA) to measure the influence of Mn(II)- and Zn(II)-specific binding on the melting temperatures (*T*_m_) of PsaA. This assay observes the overall stability of the folded state of the protein as a function of the *T*_m_. Metal-ion ligands that bind and stabilise the protein's quaternary structure induce a more thermo-stable structure, *i.e.* a higher *T*_m_. ‘As prepared’ PsaA (Fig. 1E) was found to have a major transition at 62.1°C, corresponding to the *apo*-protein, and a minor peak at 72.9°C, corresponding to the co-purifying Zn(II) form (present in ‘as prepared’ PsaA) (Table S1). This interpretation was confirmed by analysis of cation-free PsaA binding site point mutants that were purified as *apo*-proteins (Fig. S2, Table S1). Co-incubation of PsaA with saturating Zn(II) increased the overall *T*_m_ to 72.9°C (Fig. 1E, Table S2). In contrast, saturating Mn(II) only increased the *T*_m_ of the *apo*-PsaA to 65.1°C and did not affect the Zn(II)-PsaA complex. Titration of Mn(II) with PsaA showed that *apo*-PsaA demonstrated a stepwise increase in *T*_m_ (Fig. 1F, Table S2) from 63.2°C to 70.2°C, while the PsaA-Zn(II) subpopulation (72.9°C peak) was not affected by the Mn(II) additions. In conclusion, the TSA unexpectedly showed that Zn(II) induced greater thermal stability than Mn(II), despite Mn(II) having a higher affinity for PsaA.

### Extracellular Zn(II) competitively affects Mn(II) uptake in Pneumococcus

**Figure 2:**
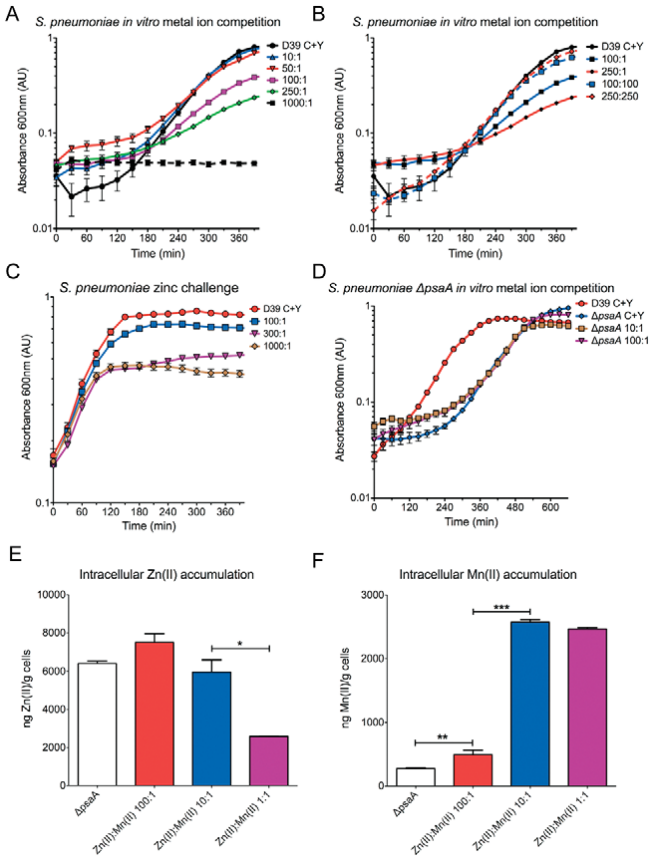
*In vitro* metal competition. *In vitro* growth measurements of *S. pneumoniae* wild-type (D39) and Δ*psaA.* (A) Bacteria were grown in C+Y medium consisting of the following Zn(II):Mn(II) ratios (in µM): 1000∶1, 250∶1, 100∶1, 50∶1 10∶1, 1∶1, and C+Y with 1 µM MnSO_4_, respectively. Data are mean (± SEM) *A*_600_ measurements from seven independent biological experiments (*n* = 7). (B) Bacteria were grown in C+Y medium consisting of the following Zn(II):Mn(II) ratios (in µM): 100∶1, 250∶1, 100∶100, 250∶250, and C+Y+1 µM MnSO_4_ supplementation, respectively. Data are means (±SEM) *A*_600_ measurements from seven independent biological experiments (*n* = 7). (C) Bacteria were grown in C+Y medium supplemented with 1 µM MnSO_4_ until an *A*_600_ of 0.3 was reached. Cells were washed in C+Y medium and then inoculated to an *A*_600_ of 0.2 in C+Y medium consisting of the following Zn(II):Mn(II) ratios (in µM): 100∶1, 300∶1, and 1000∶1. Data are means (±SEM) *A*_600_ measurements from seven independent biological experiments (*n* = 7). (D) *In vitro* growth measurements of the Δ*psaA* mutant. Bacteria were grown in C+Y medium consisting of the following Zn(II):Mn(II) ratios (in µM): 100∶1, 10∶1, and C+Y+1 µM Mn supplementation, respectively. The wild-type *S. pneumoniae* D39 grown in C+Y+1 µM Mn supplementation is shown for reference. Data are means (±SEM) *A*_600_ measurements from seven independent biological experiments (*n* = 7). (E) *S. pneumoniae* intracellular Zn(II) accumulation determined by ICPMS. Data are mean (± SEM) ng Zn(II)/g cell measurements from duplicate measurements of 2 independent biological experiments. (F) Intracellular Mn(II) accumulation determined by ICPMS. Data are mean (± SEM) ng Mn(II)/g cell measurements from duplicate measurements 2 independent biological experiments. The statistical significance of the differences in concentrations was determined by a two-tailed unpaired t-test. *P*-values of <0.05, <0.005 and <0.0005 are denoted by *, ** or ***, respectively.

We then sought to determine the phenotypic effect of Zn(II) on PsaA-mediated Mn(II) uptake. This was assessed by *in vitro* metal ion competition assays with wild-type *S. pneumoniae* and the Δ*psaA* mutant grown in the presence of differing ratios of Zn(II) in a semi-synthetic medium. Increasing the Zn(II):Mn(II) ratio slowed bacterial growth and at very high ratios, *i.e.* at 1000∶1 [1000 µM Zn(II):1 µM Mn(II)], *S. pneumoniae* growth was completely inhibited (Fig. 2A). Growth was restored, albeit heavily delayed, at Zn(II):Mn(II) ratios of 100∶1 and 250∶1, while at ratios of 50∶1 and 10∶1 Zn(II):Mn(II), *S. pneumoniae* growth was essentially the same as in the basal medium (Fig. 2A). Growth at inhibitory Zn(II) concentrations could be reversed by Mn(II) supplementation to a 1∶1 ratio relative to the Zn(II) concentration (Fig. 2B), consistent with a competitive effect of Zn(II) for PsaA. The inhibitory effect of Zn(II) was also observed on exponential phase growing cells (Fig. 2C). The addition of Zn(II) at ratios of 300∶1 and 1000∶1 inhibited growth of *S. pneumoniae* within 120 minutes (2–3 generations). Intriguingly, high ratios of Zn(II):Mn(II) slowed the growth rate of *S. pneumoniae* similar to that of the isogenic Δ*psaA* mutant.

Previously, our group has reported that the phenotypic effects of a Δ*psaA* mutant include compromised Mn(II) uptake and a slower growth rate, relative to wild-type *S. pneumoniae* [28]. As the Δ*psaA* strain lacks PsaA, and consequently has no functional high affinity Mn(II) importer, we predicted that the mutant would be insensitive to Zn(II) competition. Consistent with this hypothesis, we observed that competitive ratios of Zn(II):Mn(II), *e.g.* 250∶1 (Fig. 2D), had no effect on the already slower growth rate of the Δ*psaA* mutant.

Extracellular Zn(II) exerted a clear effect on the growth rate of *S. pneumoniae*. As this could occur through a number of possible routes in a complex biological system, we sought to determine if extracellular Zn(II) was influencing intracellular metal concentrations by conducting inductively coupled plasma mass spectroscopy (ICPMS) on *S. pneumoniae* grown in different Zn(II):Mn(II) ratios. We observed that at the competitive ratio of 100∶1, *i.e.* growth perturbing, the concentration of Zn(II) was essentially the same as that at the non-competitive 10∶1 Zn(II):Mn(II) ratio (Fig. 2E, Table S3). This indicated that despite the high concentration of extracellular Zn(II), intracellular Zn(II) homeostasis was not being disregulated. The intracellular concentrations of Zn(II) increased nearly two-fold in the wild-type when grown in the presence of high extracellular Zn(II) (10∶1 and 100∶1 Zn(II):Mn(II) ratios). Although this had no apparent physiological effect, as demonstrated by the 10∶1 Zn(II):Mn(II) ratio, the higher intracellular concentrations may render *S. pneumoniae* more susceptible to Zn(II) toxicity if Zn(II) efflux pathways were blocked or impaired. Collectively, these data support a model whereby high concentrations of extracellular Zn(II) were competitively inhibiting Mn(II) uptake and, as the intracellular Zn(II) concentration observed for the 100∶1 and 10∶1 Zn(II):Mn(II) ratios were essentially the same, it follows that the intracellular Zn(II) and hence Zn(II) toxicity was not due to toxic intracellular accumulation.

### Zn(II) is not transported via the Psa ABC permease

The lack of significant variation in the intracellular Zn(II) concentrations between the 100∶1 and 10∶1 Zn(II):Mn(II)-grown cells suggested that Zn(II) was not transported by the Mn(II) permease, despite the affinity of PsaA for Zn(II). This inference is supported by comparisons with the Δ*psaA* mutant, which lacks a Mn(II) transporter and had similar intracellular accumulations of Zn(II) to that of wild-type *S. pneumoniae* (Fig. 2E, Table S3). Taken together these data suggest a model where PsaA-Zn(II) binding results in an irreversible “dead end” complex, the physiological basis of which could be to prevent Zn(II) leakage into the bacterial cell through the Mn(II) transporter in the presence of high extracellular Zn(II) concentrations. In contrast, the Mn(II)-PsaA complex likely represents a reversible complex that, upon interaction with the ABC permease, delivers the metal ion into the transporter and allows *apo*-PsaA to be released. This model is consistent with our biochemical observations, which found that Zn(II) conferred very high thermal stabilisation on PsaA in the TSA, and that Zn(II) removal required unfolding of the protein, as it was resistant to dialysis, chelation and Mn(II) competition.

### Zn(II) competition reduces intracellular Mn(II) resulting in up-regulation of PsaBCA expression

ICPMS analysis of *S. pneumoniae* grown in the 100∶1 Zn(II):Mn(II) ratio showed a five-fold decrease in intracellular accumulation of Mn(II) (*P* = 0.002) compared to the 10∶1 and 1∶1 ratios (Fig. 2F Table S3). Thus, extracellular Zn(II) was inhibiting Mn(II) uptake by *S. pneumoniae*, thereby inducing a phenotype similar to a Δ*psaA* mutant. However, unlike the mutant strain, the 100∶1 Zn(II):Mn(II)-grown *S. pneumoniae* had an intact high affinity Mn(II) transporter. The residual Mn(II) uptake observed in the Δ*psaA* mutant most likely occurs via non-specific transport.

We then sought to determine what effect competitive ratios of Zn(II) had on PsaBCA expression. The expression of PsaBCA is regulated by PsaR, a DtxR family regulator, which represses *psaBCA* transcription in response to Mn(II) while de-repression occurs at low Mn(II) or high Zn(II) concentrations [37]. Under our experimental conditions, 100∶1 Zn(II):Mn(II)-grown *S. pneumoniae* demonstrated slower growth and decreased intracellular Mn(II) compared to 10∶1 Zn(II):Mn(II)-grown *S. pneumoniae*, which had unaffected growth. An analysis of *psaA* transcription and *psaA* expression found that, in 100∶1 Zn(II):Mn(II)-grown cells, both were highly up-regulated relative to the 10∶1 grown cells (Fig. S3A and S3B). As the intracellular Zn(II) concentrations in both the 100∶1 and 10∶1 Zn(II):Mn(II)-grown *S. pneumoniae* were essentially the same, the observed PsaR-dependent up-regulation of expression of the *psaBCA* operon would be most likely due to the Mn(II) starvation phenotype and similar to that previously observed in the Δ*psaA* strain [37].

### Zn(II) competition heightens sensitivity of *S. pneumoniae* to oxidative killing

Mn(II) restriction in *S. pneumoniae* has two well established consequences, namely a reduction in oxidative stress response capability and a concomitant loss of *in vivo* virulence [23], [28], [29]. Therefore, to assess whether the Zn(II)-dependent inhibition of Mn(II) uptake was inducing a Δ*psaA*-like phenotype, we investigated the capacity of 100∶1 Zn(II):Mn(II)-grown *S. pneumoniae* to tolerate oxidative stress mediated chemically, via paraquat or by human polymorphonuclear leucocytes (PMNs).

**Figure 3:**
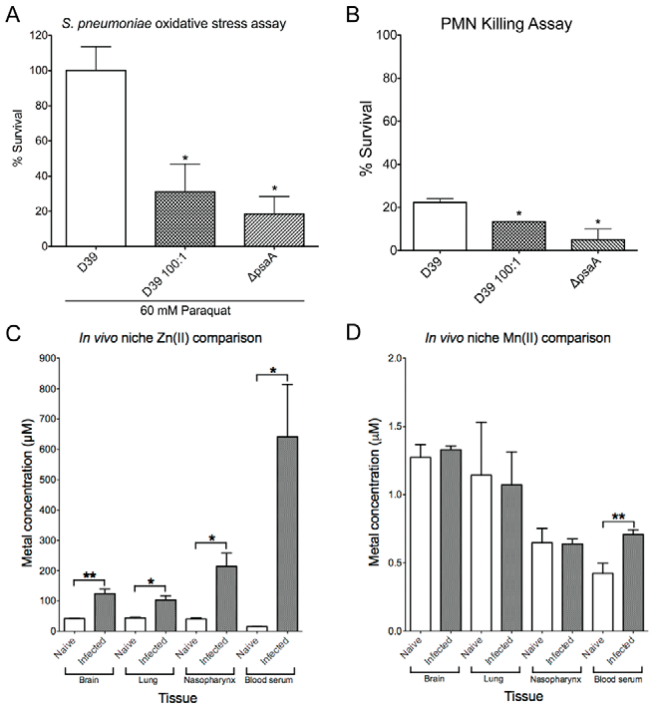
*In vitro* effects on bacterial survival and *in vivo* metal concentrations. (A) Paraquat killing of the *S. pneumoniae* wild-type (D39) and Δ*psaA* mutant grown in C+Y+1 µM Mn, and *S. pneumoniae* grown in 100 µM Zn(II)∶1 µM Mn(II) (D39 100∶1). Survival was calculated as a percentage of colonies at 30 minutes compared to 0 minutes. The experiment was performed with 3 independent biological samples (*n* = 3) and data are the means (±SEM). (B) PMN killing of *S. pneumoniae* D39 and Δ*psaA* mutant grown in C+Y+1 µM MnSO_4_, and *S. pneumoniae* grown in 100 µM Zn(II):1 µM Mn(II) (D39 100∶1). The experiment was performed in triplicate (*n* = 3) and shown data are means (±SEM). (C) *In vivo* niche Zn(II) comparisons. Zn(II) accumulation determined by ICPMS from mouse tissues of naïve (*n* = 5) and *S. pneumoniae*-infected mice (*n* = 10). The statistical significance of the differences in the *in vivo* mouse tissue Zn(II) concentrations was determined by a two-tailed unpaired t-test. (D) *In vivo* niche Mn(II) comparisons. Mn(II) accumulation determined by ICPMS from mouse tissues of naïve (*n* = 5) and *S. pneumoniae*-infected mice (*n* = 10). The statistical significance of the differences was determined by a two-tailed unpaired t-test. *P*-values of <0.05, <0.005 and <0.0005 are denoted by *, ** or ***, respectively.

Paraquat is a redox compound that generates superoxide in the cytoplasm, and survival after exposure requires effective oxidative stress management [23]. After 30 min. of paraquat exposure both the 100∶1 Zn(II):Mn(II)-grown *S. pneumoniae* and the Δ*psaA* strain had significantly lower survival rates (31% [*P* = 0.046] and 18% [*P* = 0.040], respectively) when compared to the Mn(II) replete wild-type *S. pneumoniae* (100%) (Fig. 3A). Following the hypothesis that reduced Mn(II) accumulation results in hypersensitivity to oxidative stress, we next examined whether this heightened susceptibility to killing extended to human PMNs (Fig. 3B). Both the 100∶1 Zn(II):Mn(II)-grown *S. pneumoniae* and the Δ*psaA* strain had significantly lower survival rates after incubation with PMN (13.4% [*P* = 0.0292] and 5.0% [*P* = 0.0285], respectively) when compared to the Mn(II) replete wild-type *S. pneumoniae* (22.3%). The increased susceptibility of the Δ*psaA* mutant and the 100∶1 Zn(II):Mn(II) grown wild-type *S. pneumoniae* demonstrates that Mn(II) has a direct role in resisting PMN killing. Taken together these data show that Zn(II) competitively inhibits intracellular Mn(II) acquisition in *S. pneumoniae* leading to significantly increased susceptibility to oxidative stress, similar to the Δ*psaA* strain.

### Infection by *S. pneumoniae in vivo* elicits an increase in the Zn(II):Mn(II) ratio

The physiological consequences of Mn(II) deprivation, as a result of Zn(II) competition *in vitro*, suggested that *S. pneumoniae* survival *in vivo* could be compromised, similar to the Δ*psaA* strain. Therefore, we investigated the levels of Mn(II) and Zn(II), and other cations, in tissue samples collected from naïve and *S. pneumoniae*-infected mice. For naïve mice, the ratios of Zn(II) to Mn(II) in niches that would be subject to colonization by *S. pneumoniae* did not exceed ∼60∶1 (Table 1). Thus, the observed ratios of Mn(II) to Zn(II) within these physiological niches should be permissive for Mn(II) acquisition by PsaBCA. For mice infected with *S. pneumoniae*, the samples harvested 48 hours post-infection showed significantly shifted metal ratios, with Zn(II) concentrations increasing in all tissues (Table 1, Fig. 3C). This effect was largely specific for Zn(II), although minor increases were observed with Fe(II) in the nasopharynx and the brain and Cu(II) in blood serum (Table S4). The increase of Zn(II) was most dramatic in blood serum and the nasopharynx, where the Zn(II):Mn(II) ratio increased to approximately 900∶1 (*P* = 0.0292) and 330∶1 (*P* = 0.0163), respectively. The lack of variation among the majority of other metal ions examined (Table S4) indicated that elevation of Zn(II) has a major role in host response to *S. pneumoniae* infection.

**Table 1:** Physiological ratios of tissue metal concentrations.

	Zn(II)/Mn(II) Ratio		
Sample	Naïve (*n* = 5)	Infected (*n* = 10)	Fold Change*
Brain	33	93	2.8
Lung	39	96	2.4
Nasopharynx	63	336	5.3
Blood serum	37	905	24.4

*The statistical significance of the differences between the metal ion concentrations before (naïve) and after (infected) infection for all samples were analyzed using a two-tailed unpaired t-test. *P*-values for all analyses were at least <0.05.

These findings were consistent with the earlier study of Strand and co-workers [38], which found that Zn(II)-deficient mice had heavier *S. pneumoniae* nasopharyngeal colonization, compared to the Zn(II) replete mice, and only Zn(II) deficient mice had *S. pneumoniae* bacteremia. The dramatic elevation of Zn(II) observed in these two tissues, upon infection with *S. pneumoniae* in our study, suggested that Zn(II) could compete with Mn(II) (which remained largely unchanged) for PsaA interaction (Table 1, Fig. 3D) and, therefore, demonstrate that metal ions have a role in ameliorating bacterial propagation and dissemination *in vivo*.

## Discussion

Although nearly 30% of all proteins contain metal ions [21], an excess of certain transition metal ions can exert significant toxicity. The potential roles of such metals in resisting bacterial invasion are increasingly being recognized. Notably the importance of copper in macrophages for bactericidal activity has recently been demonstrated [39]. Our present study provides the first direct evidence that extracellular Zn(II) can exert its toxicity towards *S. pneumoniae* by competing with Mn(II) for binding to the PsaA solute binding protein, thereby preventing the acquisition of Mn(II) via the Psa permease. While our study was being completed, Jacobsen and co-workers similarly proposed that Zn(II) toxicity results in cytoplasmic Mn(II) deficiency, but no evidence for the mechanism was provided [40]. We have shown that Zn(II) competition results in reduced Mn(II) accumulation and elicits a phenotype nearly identical that of an isogenic Δ*psaA* mutant. Reduced intracellular Mn(II) perturbs bacterial growth as the capacity to manage oxidative stress, which occurs during aerobic growth, is compromised [23], [28]. This is particularly noticeable during the outgrowth of stationary phase cells, which showed impaired growth at Zn(II):Mn(II) ratios of 100∶1 and greater, while exponential phase growing cells were inhibited at higher ratios, presumably as Mn(II) was titred out by cell division. This may also indicate that Mn(II) has distinct intracellular roles at different stages of *S. pneumoniae* growth. Very high (>1 mM) concentrations of zinc completely bacterial inhibited growth and it is likely that under these conditions Zn(II) toxicity was mediated by mechanisms in addition to competition for PsaA. Our findings also provide a physiological rationale for the Zn(II)-dependent regulation of PsaR [37] as a mechanism for regulating expression of the Mn(II) transporter during exposure to high levels of the competing cation. Cluster A-1 SBPs are present in a broad range of pathogenic bacteria, such as *S. pneumoniae, Yesinia pestis*, *Staphyloccocus aureus*, and *S. pyogenes* [19], [31], [32], [41], and so the competitive mechanism of Zn(II) toxicity proposed for *S. pneumoniae* could also extend to these bacteria.

This investigation also resolves an area of significant confusion as to the presence of Zn(II) in the original crystal structure of PsaA [18], which has remained contradictory to all of the physiological data for the role of the Psa permease in *S. pneumoniae*. Here we show PsaA in complex with its physiological ligand and the difference in the affinities for Mn(II) and Zn(II) confirm its physiological role in Mn(II) acquisition. Our biophysical characterization also provides an explanation for the presence of Zn(II) in the original crystal structure. The thermal stabilization data show that Zn(II) binding induces a highly stabilized from of PsaA and it is highly likely that this would preferentially crystallize in comparison to the less thermally stable *apo*- form of the protein. This is also consistent with the behaviour of other Cluster A-1 Mn(II)-SBPs, all of which show a preference for Mn(II) but still retain an interaction with Zn(II), albeit at a lower affinity. Furthermore, consistent with the lack of solvent access to the binding site in the metal loaded-PsaA structures, we observed that Zn(II) could not be extracted from PsaA by chelating agents, despite the protein's lower affinity for the metal, or by competition with Mn(II). This supports our observations that, at high ratios, Zn(II) binding to PsaA could effectively inactivate the Psa permease leading to impairment of Mn(II) uptake as shown in our *in vitro* studies.

Our study also highlights the importance of Zn(II) during *in vivo* infection and implicates a role for Zn(II) in the host immune response. Dietary zinc deficiency is a global health issue that affects 2 billion people [42] and, in developing countries, zinc deficiency is associated with a higher burden of respiratory disease [4], [43]. In recent years Zn(II) supplementation therapies have proven to be efficacious in reducing the incidence of respiratory infections [4], [5], [12], [43]. These studies have also shown that inadequate dietary Zn(II) can reduce mean blood serum Zn(II) concentrations by up to 76%. Thus, dietary Zn(II)-deficiency can significantly reduce the Zn(II):Mn(II) ratio in the blood and, presumably, other tissues [4], [5], [43].

The association between Zn(II) and optimal immune function is well known and our findings offer a plausible molecular explanation for the protective role of zinc. We have shown that Zn(II) concentrations were significantly elevated within 48 hours of bacterial infection in all niches colonized by *S. pneumoniae,* consistent with the importance of Zn(II) in the innate immune response. Furthermore, a comparison between the naïve and infected states clearly shows that the alterations in metal ion concentrations in host niches were essentially Zn(II)-specific with only isolated changes observed for other trace elements. We propose that it is likely that the observed increase in Zn(II) concentration *in vivo* is due to extracellular release of Zn(II) to recruit immune effector cells [44], [45] and to compete for Mn(II) acquisition. This inference is consistent with the massive elevation of the Zn(II):Mn(II) ratio in the nasopharynx and in blood serum, two niches that were hyper-susceptible to *S. pneumoniae* colonization in Zn(II)-deficient mice [38]. Furthermore, our observations are also consistent with the recent findings of Corbin and co-workers [13], who found that the Gram-positive pathogen *Staphylococcus aureus* had a similar dependence on Mn(II) in abscess tissue, where the host protein calprotectin was identified as mediating *in vivo* Mn(II) restriction. Assessing *in vivo* metal ion bioavailability remains a technical challenge, since even in blood serum, metal ions may be sequestered in proteins and other carrier molecules. Despite this caveat, which applies to this study and others such as Corbin and co-workers [13], our data directly implicate the specific elevation of Zn(II) as a major factor in host response to infection.

Based on the available data we propose that, during *in vivo* infection, Mn(II) bioavailability could be restricted due to elevation of Zn(II):Mn(II) ratio by the release of Zn(II) from damaged or apoptotic cells and from sequestering proteins such as metallothionein. This would initially slow *S. pneumoniae* growth while also increasing its susceptibility to oxidative killing, as it is the ratio, not the absolute concentration of the two metal ions, that is important. This would be consistent with the requirement of Mn(II) uptake for *S. pneumoniae* infection *in vivo*, as the Δ*psaA* mutant is totally avirulent [27], [28], [29]. Zn(II) was released during infection at high ratios relative to Mn(II) in the nasopharynx and in blood serum. In our *in vitro* studies, these ratios were inhibitory and it is not unreasonable to speculate that these ratios *in vivo* could obfuscate Mn(II)-PsaA interactions and thereby induce a Mn(II)-starvation phenotype in the invading pathogen. The host release of Zn(II) would have the additional benefit of also driving recruitment of leukocytes and other components of the innate immune response [44], [45]. The cumulative effect of this would be that elevation of the Zn(II):Mn(II) ratio *in vivo* would also increase the susceptibility of *S. pneumoniae* to oxidative killing by immune effector cells, such as PMNs, as we have shown *in vitro*, thereby facilitating more efficient defence against the invading pathogen.

PMNs are a major component of the innate response and in addition to their oxidative killing mechanisms they contain large stores, up to 50% of their total protein, of the metal binding protein calprotectin [46]. Corbin and co-workers [13] first postulated that calprotectin could be released and directed at bacterial pathogens as a mechanism for restricting metal ion availability *in vivo.* Supporting this inference, they observed that calprotectin-deficient mice had increased abscess formations and bacterial burdens relative to infected wild-type animals. Release of calprotectin by PMNs during the immune response could also have a significant effect on the Zn(II):Mn(II) ratio, by sequestering Mn(II). This would increase the susceptibility of invading bacteria to oxidative killing mechanisms, consistent with both the earlier suggestions of Corbin and co-workers [13] and in our model for *in vivo* Mn(II) starvation of the invading bacterium.

Collectively, this study demonstrates the biophysical basis of metal specificity for an essential bacterial cell-surface protein and its importance for bacterial propagation. We have provided direct evidence of how the usually non-toxic metal ion Zn(II) mediates toxicity to *S. pneumoniae,* namely by competition for Mn(II) acquisition leading to intracellular Mn(II) starvation. Furthermore, we have provided a molecular explanation for how this could be harnessed and exploited by the innate immune system to heighten the bacterium's sensitivity to immune effector cells. This has great significance in the context of the nutritional importance of Zn(II) and its association with optimal immune function.

## Materials and Methods

### Ethics statement

All procedures performed in this study were conducted with a view to minimising the discomfort of the animals, and used the minimum numbers to generate reproducible and statistically significant data. All experiments were approved by the University of Adelaide Animal Ethics Committee (Animal Welfare Assurance number A5491–01; project approval number S-2010–001) and were performed in strict adherence to guidelines dictated by the Australian Code of Practice for the Care and Use of Animals for Scientific Purposes.

### Expression and purification of wild-type and mutant derivatives of PsaA

High-level expression and purification was conducted as described previously [47]. PsaA was enriched for Mn(II) by harvesting cells and purification in the presence of 5 mM MnSO_4_. SDS-PAGE and western blotting was conducted as described previously [28].

### *Apo*-PsaA generation and ICPMS

Demetallated (*apo*) PsaA was prepared from Mn(II)-enriched PsaA (50 µM) that was heated to 62.5°C in the presence of 0.5 mM EDTA for 5 minutes. The sample was then cooled for 10 min, centrifuged and 18,000 × *g* for 10 min and the supernatant desalted on a PD10 column (GE Healthcare). The sample was analyzed for Zn(II) and Mn(II) content by boiling 10 µM protein at 95°C for 20 min in 3.5%, 7% or 14% HNO_3_. Samples were analyzed on an Agilent 7500cx ICP-MS (Adelaide Microscopy, University of Adelaide). Routine treatment used 3.5% HNO_3_ as no differences were found in the quantity of metal released. *Apo*-PsaA was defined as having less than 10% total metal content on a mol metal/mol PsaA basis.

### Construction and cloning of Glu205→Gln and Asp280→Asn mutants of PsaA

Point mutants were constructed by overlap extension PCR, essentially as described previously [48]. The mutants were confirmed to be in-frame by PCR and sequence analysis using primers flanking each gene in question. The primers used for construction and validation of the mutants are listed in Table S5. Overlap extension products were then cloned, in frame, into the respective restriction sites in pQE30 (Qiagen, Germany).

### Biophysical analyses

ITC was performed using a VP-ITC unit (Microcal, USA) with *apo-*PsaA, 4.5, 10, 15 or 20** µ**M, in 20****mM sodium phosphate buffer, pH 6.5 at 25°C. Samples were centrifuged and degassed prior to analysis. MnSO_4_ was injected at 3****min intervals with 56 injections of 4** µ**l with a 10 second injection time. ZnSO_4_ was injected at 3****min intervals with 28 injections of 10** µ**l with a 20 second injection time. A stir rate of 307 per min was used in both experiments. Data were analyzed using the Origin 7.0 software (Microcal) and the parameters (± SEM) determined. The thermal shift assay was based on the Thermfluor assay [49] conducted using 10** µ**M PsaA in 100****mM MOPS, pH 7.2, 150****mM NaCl, 5× SYPRO Orange (Invitrogen) in the presence of 10** µ**M to 1000** µ**M metal, using a Roche LC480 Real-Time Cycler. For further details see Text S3.

### *In vitro* growth measurements

Frozen stock *S. pneumoniae* D39 were prepared as described previously [28]. For *in vitro* growth measurements, frozen stock culture was added to C+Y with 1 µM MnSO_4_ and supplemented with ratios of metal ions as specified. The starting *A*_600_ was 0.05 for all cultures. For the Zn(II) challenge experiment a frozen stock culture was added to C+Y with 1 µM MnSO_4_ to a starting *A*_600_ of 0.05 and grown to an *A*_600_ of 0.3. Cells were washed with C+Y, pre-warmed to 37°C, and then reinoculated into C+Y with 1 µM Mn SO_4_ and supplemented with 100 µM, 300 µM or 1 mM ZnSO_4_, to an *A*_600_ of 0.2. Cell growth was then monitored at *A*_600_. All analyses were carried out in at least biological triplicate. Bacteria prepared in this manner were washed twice, each in 10 volumes of PBS, before western blotting. For ICPMS analyses cells were washed 3 times, 14,000 *x g* for 10 min, in PBS +5 mM EDTA and then washed 3 times with PBS. The dry cell mass was determined and the material boiled at 95°C for 20 min in 3.5%, 7% or 14% HNO_3_. The metal-ion containing supernatant was collected by centrifugation at 14,000 *x g* for 30 min and metal content determined on an Agilent 7500cx ICPMS (Adelaide Microscopy, University of Adelaide). Routine treatment used 3.5% HNO_3_ as no differences were found in the quantity of metal released.

### *In vivo* metal content determination

Outbred 5–6 week old female CD1 (Swiss) mice were used in these experiments, under the approval of the Animal Ethics Committee of The University of Adelaide. For metal content determination in naïve mice (*n* = 5), samples of nasopharynx, lungs, blood and brain were harvested as described previously [50], using PBS only (without trypsin or EDTA). For metal content determination in mice challenged with pneumococci (*n* = 10) were infected intranasally with approx. 10^7^ c.f.u. of *S. pneumoniae* D39, following the procedures described previously [50]. Mice were sacrificed at 48 hr-post-infection and samples of nasopharynx, lungs, blood and brain of each mouse were harvested and processed as described previously [50], again using PBS only. Tissue samples were analyzed for mass, except blood serum, and boiled in 3.5% HNO_3_ for 20****min at 95°C. Blood serum was diluted in 7% HNO_3_ and boiled for 20 min at 95°C. The metal-ion containing supernatant was collected by centrifugation at 14,000 *x g* for 30 min and metal content determined on an Agilent 7500cx ICPMS (Adelaide Microscopy, University of Adelaide). The tissue concentrations of metal ions were calculated using the mass or volume of tissue dissolved in HNO_3_.

### Real-time RT-PCR

One-step relative quantitative real time RT-PCR using a Roche LC480 Real-Time Cycler, was performed as described previously [48]. The *psaA* primers are listed in Table S4 and were used at a final concentration of 200 nM per reaction. 16S rRNA was employed as a control. Amplification data were analyzed using the comparative critical threshold (2^−ΔΔCT^) method [48].

### Crystallization, data collection and structure determination

Manganese-enriched IMAC-purified protein was further purified on a HiLoad 26/60 column. The protein was eluted in 0.2 M MnSO_4_, 0.2 M NaCl and 20 mM HEPES/NaOH, pH 7.5 at 20°C. The peak-containing fractions were analyzed for purity by SDS-PAGE and selected fractions were concentrated to 18 mg.ml^−1^ using Amicon Ultra-4 centrifugal devices (Millipore). The crystals of the Mn(II)-bound form were optimized by several rounds of microseeding utilizing the nuclei of Mn(II)-bound PsaA crystals obtained by vapour diffusion in hanging drops at 18°C essentially as conducted previously [18]. The seed was introduced into a drop equilibrated overnight and containing 1 µl of protein and 1 µl of reservoir solution containing 33% PEG 1500, 0.15 M SPG buffer pH 4.0, 0.2 M MnSO_4_ suspended over 0.5 ml of reservoir solution. SPG buffer was produced by mixing succinic acid, sodium dihydrogen phosphate, and glycine in the molar ratios 2∶7∶7 and pH adjusted by adding 10 N NaOH. Crystals grew to the maximum size of 200 µm x 200 µm x 100 µm within 7–10 days. For data collection, the crystals were cryoprotected with 20% glycerol before being flash-cooled by rapid immersion in liquid nitrogen. The diffraction data were collected on a single crystal on the MX2 microfocus beamline of the Australian Synchrotron [51] (Melbourne, Australia) operating at 6545 eV (for details and refinement statistics see Table S6). For further details of the data collection and analysis see Text S3.

### Bacterial killing assays

Bacteria were grown to an *A*_600_ = 0.3 in minimal media with or without Zn(II) supplementation, washed 3 times with PBS +2.5 mM EDTA to remove excess cations and then 3 times with PBS. Cells were incubated for 30 minutes with 60 mM paraquat (Sigma-Aldrich) and then serially diluted and plated on blood-agar. Plates were incubated overnight at 37°C +5% CO_2_. Survival was calculated as a percentage of colonies at 30 minutes compared to 0 minutes. The PMN killing assay was adapted from [52], and further details are provided in Text S3.

## Supporting Information

Figure S1 **PsaA-Mn(II) structural comparisons.** (A) PsaA-Mn(II) (PDB ID: 3ZTT) overlayed with MntC-Mn(II) (PDB ID: 1XVL). Superposition was performed using COOT (see Text S2 for further details) using the SSM structural alignment function (34.2% sequence identity; RMSD = 1.31 Å for 266 Cα atoms). PsaA shown in green and MntC is in blue. The metal binding site residues are also shown and the Mn(II) ions are shown as purple spheres. (B) The metal binding site from S1A is shown in more detail, with the MntC residues and Mn(II) labels italicised. (C) PsaA-Mn(II) (green) overlayed with PsaA-Zn(II) (blue) using COOT (RMSD = 0.496 Å for Cα atoms). (D) The metal binding site from S1*C* is shown in more detail. The manganese ion is shown as a purple sphere and zinc ion is shown as an orange sphere. The imidazole ring of His-139 is rotated 31 degrees to accommodate the larger atomic volume of the Mn(II) atom in the PsaA-Mn(II) structure but this has no effect on the metal ion distance to the N^ε2^ ligand.
(TIF)

Figure S2 **Thermal stability of PsaA mutants.** The thermal unfolding of the protein was followed by the presence of the SYPRO Orange fluorescent probe. The samples were pre-incubated for 10 minutes with the indicated metal ion concentration and then subjected to thermal unfolding from 25°C to 97°C at a heating rate of 1°C per minute. The normalized inverse plot of the first derivative of the fluorescence over temperature allows for accurate determination of the *T*_m_ after background subtraction. The sets of curves are representative of three independent experiments. (A) PsaA Gln205 with saturating concentrations of Mn(II) or Zn(II) (100-fold excess). (B) PsaA Asn280 with saturating concentrations of Mn(II) or Zn(II) (100-fold excess).
(TIF)

Figure S3 ***In vitro*** **metal competition effect on** ***psaA*** **expression.** (A) Western blot analysis of lysates of *S. pneumoniae* D39 grown in C+Y medium consisting of the following Zn(II):Mn(II) ratios: 100∶1, 10∶1, 1∶1, respectively. Blots are from two biological replicates for each growth condition. (B) *psaA* gene mRNA concentrations from *S. pneumoniae* D39 grown in C+Y medium consisting of different Zn(II):Mn(II) ratios, relative to concentrations obtained from Zn(II):Mn(II) (1∶1) ratio. Real-time RT-PCR data for the indicated conditions were normalized against those obtained for the 16S rRNA control. Quantitative fold differences for the *psaA* transcript were determined using the 2^-ΔΔ*C*^_T_ method^30^ **.** Data are means (± SEM) of duplicate reactions from two biological replicates.
(TIF)

Table S1 **Purified PsaA Metal Content (mol metal/mol protein).**
(DOC)

Table S2 **Thermal stability of 10 µM PsaA at different metal ion concentrations.**
(DOC)

Table S3 ***S. pneumoniae*** **metal ion competition.**
(DOC)

Table S4 ***In vivo*** **niche metal concentrations.**
(DOC)

Table S5 **Oligonucleotide primers used in this study.**
(DOC)

Table S6 **PsaA-Mn(II) structure data collection and refinement statistics.**
(DOC)

Text S1 **Isothermal calorimetric analysis of PsaA.** Interpretation of the ITC analysis of PsaA presented in Fig. 1A and B.
(DOC)

Text S2 **Crystallographic analysis of PsaA-Mn(II).** Interpretation of the PsaA-Mn(II) crystal structure presented in Figs. 1C, D, and S1.
(DOC)

Text S3 **Supporting Methods and References.**
(DOC)
