# Supplementary figures and images for: A Molecular Mechanism for Bacterial Susceptibility to Zinc
Source: PLoS Pathog. 2011 Nov 3;7(11):e1002357. doi: 10.1371/journal.ppat.1002357 (PMC3207923; doi:10.1371/journal.ppat.1002357)

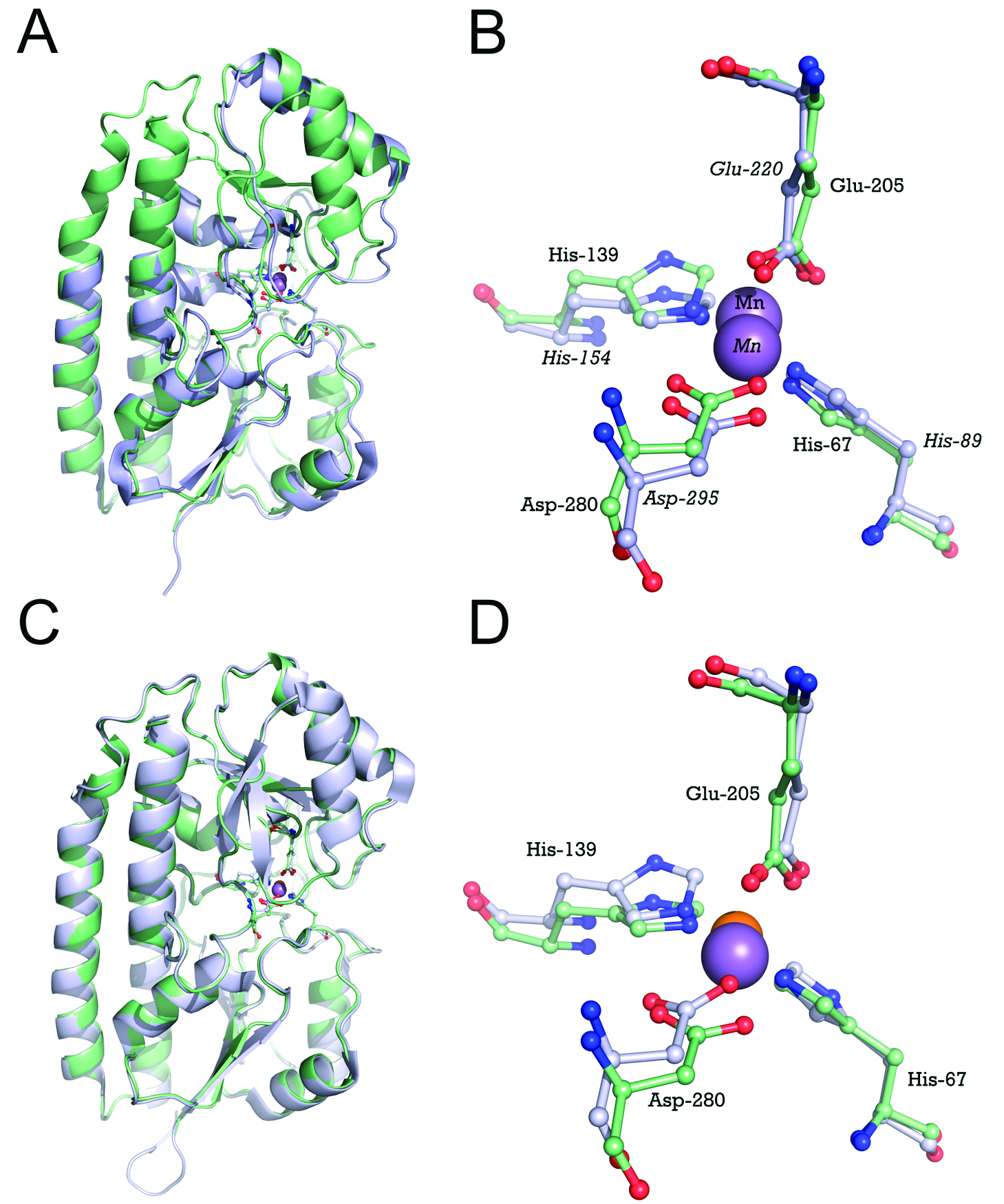

Supplement: Figure S1 — PsaA-Mn(II) structural comparisons. (A) PsaA-Mn(II) (PDB ID: 3ZTT) overlayed with MntC-Mn(II) (PDB ID: 1XVL). Superposition was performed using COOT (see Text S2 for further details) using the SSM structural alignment function (34.2% sequence identity; RMSD = 1.31 Å for 266 Cα atoms). PsaA shown in green and MntC is in blue. The metal binding site residues are also shown and the Mn(II) ions are shown as purple spheres. (B) The metal binding site from S1A is shown in more detail, with the MntC residues and Mn(II) labels italicised. (C) PsaA-Mn(II) (green) overlayed with PsaA-Zn(II) (blue) using COOT (RMSD = 0.496 Å for Cα atoms). (D) The metal binding site from S1C is shown in more detail. The manganese ion is shown as a purple sphere and zinc ion is shown as an orange sphere. The imidazole ring of His-139 is rotated 31 degrees to accommodate the larger atomic volume of the Mn(II) atom in the PsaA-Mn(II) structure but this has no effect on the metal ion distance to the Nε2 ligand. (TIF) [file ppat.1002357.s001.tif]

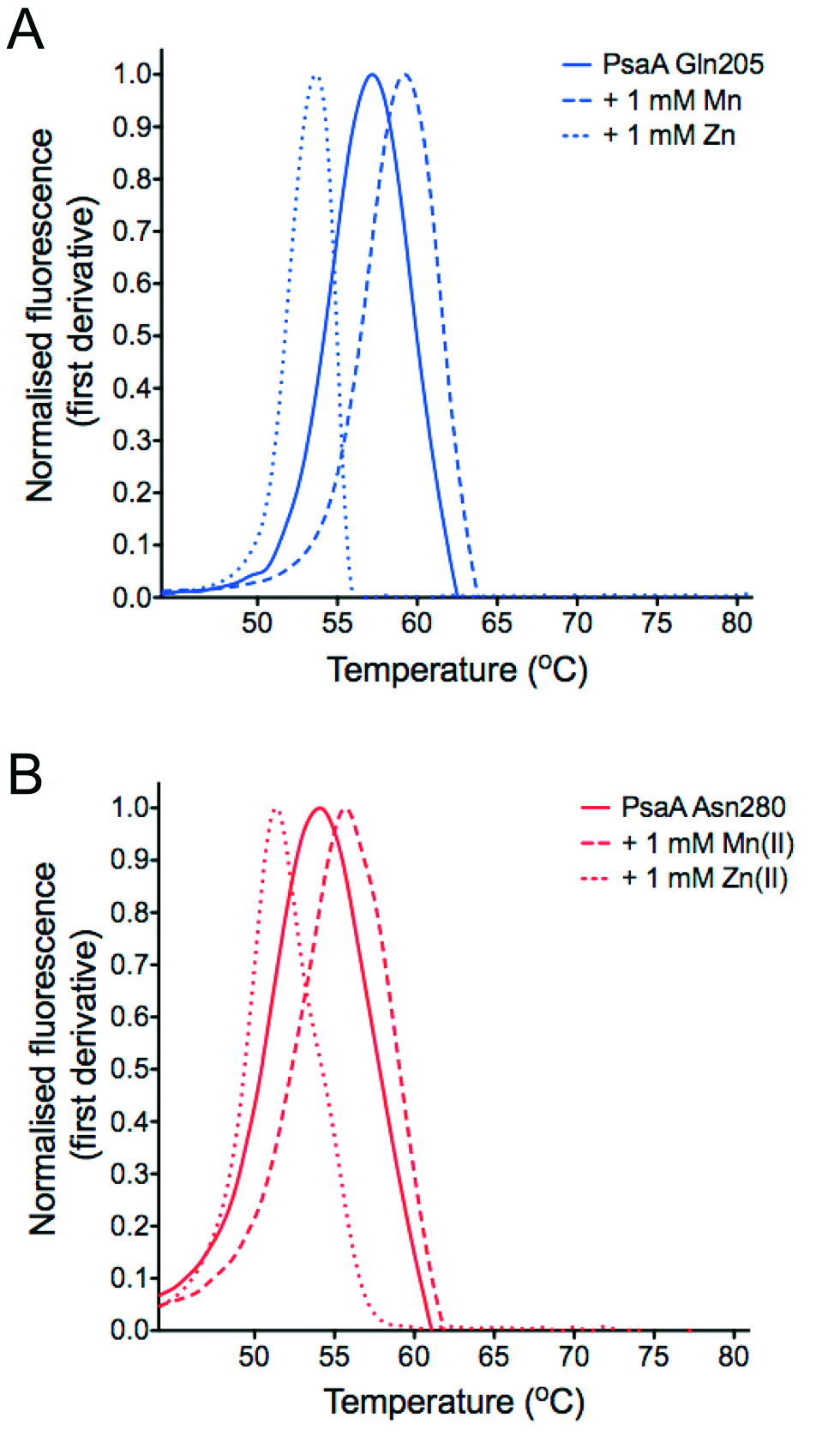

Supplement: Figure S2 — Thermal stability of PsaA mutants. The thermal unfolding of the protein was followed by the presence of the SYPRO Orange fluorescent probe. The samples were pre-incubated for 10 minutes with the indicated metal ion concentration and then subjected to thermal unfolding from 25°C to 97°C at a heating rate of 1°C per minute. The normalized inverse plot of the first derivative of the fluorescence over temperature allows for accurate determination of the T m after background subtraction. The sets of curves are representative of three independent experiments. (A) PsaA Gln205 with saturating concentrations of Mn(II) or Zn(II) (100-fold excess). (B) PsaA Asn280 with saturating concentrations of Mn(II) or Zn(II) (100-fold excess). (TIF) [file ppat.1002357.s002.tif]

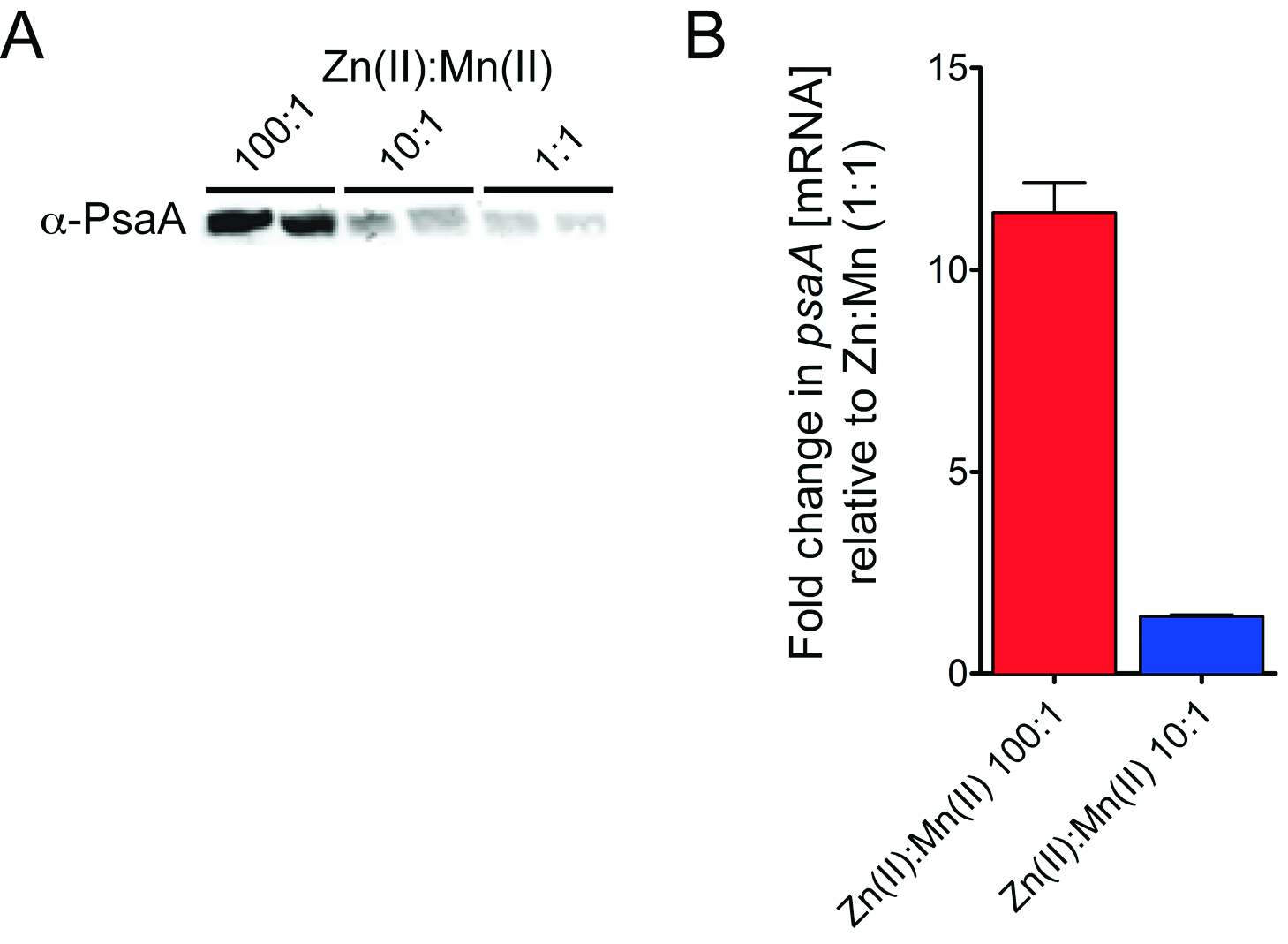

Supplement: Figure S3 — In vitro metal competition effect on psaA expression. (A) Western blot analysis of lysates of S. pneumoniae D39 grown in C+Y medium consisting of the following Zn(II):Mn(II) ratios: 100∶1, 10∶1, 1∶1, respectively. Blots are from two biological replicates for each growth condition. (B) psaA gene mRNA concentrations from S. pneumoniae D39 grown in C+Y medium consisting of different Zn(II):Mn(II) ratios, relative to concentrations obtained from Zn(II):Mn(II) (1∶1) ratio. Real-time RT-PCR data for the indicated conditions were normalized against those obtained for the 16S rRNA control. Quantitative fold differences for the psaA transcript were determined using the 2-ΔΔC T method30 . Data are means (± SEM) of duplicate reactions from two biological replicates. (TIF) [file ppat.1002357.s003.tif]
