# Supplementary material for: A Molecular Mechanism for Bacterial Susceptibility to Zinc
Source: PLoS Pathog. 2011 Nov 3;7(11):e1002357. doi: 10.1371/journal.ppat.1002357 (PMC3207923; doi:10.1371/journal.ppat.1002357)
Supplement: Table S1 — Purified PsaA Metal Content (mol metal/mol protein). (DOC) [file ppat.1002357.s004.doc]

**Table S**1. Purified PsaA Metal Content (mol metal/mol protein)

| **PsaA Isoform** | **Mn(II) As Purified** | **Zn(II) As Purified** | **Mn(II) Saturation** | **Zn(II) Saturation** |
| --- | --- | --- | --- | --- |
| WT | 0.00 | 0.35 | 1.24 | 5.40 |
| Glu205→Gln | 0.00 | 0.05 | 0.95 | 1.06 |
| Asp280→Asn | 0.01 | 0.04 | 0.12 | 2.49 |
