# Supplementary material for: A Molecular Mechanism for Bacterial Susceptibility to Zinc
Source: PLoS Pathog. 2011 Nov 3;7(11):e1002357. doi: 10.1371/journal.ppat.1002357 (PMC3207923; doi:10.1371/journal.ppat.1002357)
Supplement: Table S2 — Thermal stability of 10 µM PsaA at different metal ion concentrations. (DOC) [file ppat.1002357.s005.doc]

**Table S2. Thermal stability of 10 **M PsaA at different metal ion concentrations

| **Metal Concentration** | **Mn(II)-*T*_m_** | **Zn(II)-*T*_m_** |
| --- | --- | --- |
| Buffer only | 62.1°C | 62.1°C |
| 1 μM | 63.2°C | 63.1°C |
| 10 μM | 65.1°C | 72.9°C |
| 100 μM | 67.0°C | 72.9°C |
| 1 mM | 70.2°C | 74.0°C |
