# Supplementary material for: A Molecular Mechanism for Bacterial Susceptibility to Zinc
Source: PLoS Pathog. 2011 Nov 3;7(11):e1002357. doi: 10.1371/journal.ppat.1002357 (PMC3207923; doi:10.1371/journal.ppat.1002357)
Supplement: Table S3 — S. pneumoniae metal ion competition. (DOC) [file ppat.1002357.s006.doc]

**Table S3. *S. pneumoniae*** metal ion competition

| **Ratio** | **Mn(II) (ng/g cells)** | **Zn(II) (ng/g cells)** |
| --- | --- | --- |
| Δ*psaA* C+Y 1 μM Mn(II) | 275.2 ± 16 | 6413.6 ± 606 |
| D39 C+Y 1 μM Mn(II) | 2463.1 ± 26 | 3400.6 ± 27 |
| D39 100:1 Zn(II):Mn(II) | 493.9 ± 72 | 7503.7 ± 457 |
| D39 10:1 Zn(II):Mn(II) | 2466.4 ± 22 | 5961.8 ± 628 |
| D39 1:1 Zn(II):Mn(II) | 2573.9 ± 41 | 2583.5 ± 12 |
