# Supplementary material for: A Molecular Mechanism for Bacterial Susceptibility to Zinc
Source: PLoS Pathog. 2011 Nov 3;7(11):e1002357. doi: 10.1371/journal.ppat.1002357 (PMC3207923; doi:10.1371/journal.ppat.1002357)
Supplement: Table S4 — In vivo niche metal concentrations. (DOC) [file ppat.1002357.s007.doc]

**Table S4 *In vivo*** niche metal concentrations

| **Niche** | **Naïve Concentration (μM ±** **SEM) (n=5)** | **Infected Concentration (μM ±** **SEM) (n=10)** | **Fold Change**  **(Infected/Naïve)** | **Statistical Significance^a^** |
| --- | --- | --- | --- | --- |
| Blood Mn(II) | 0.42 ± 0 .07 | 0.71 ± 0.03 | 1.67 | *; P = 0.0014 |
| Brain Mn(II) | 1.27 ± 0.09 | 1.33 ± 0.03 | 1.04 | n.s. |
| Lung Mn(II) | 1.14 ± 0.39 | 1.07 ± 0.24 | 0.94 | n.s. |
| Nasopharynx Mn(II) | 0.65 ± 0.11 | 0.64 ± 0.04 | 0.99 | n.s. |
| Blood Fe(II) | 138.37 ± 9.15 | 256.75 ± 53.63 | 1.86 | n.s. |
| Brain Fe(II) | 26.75 ± 2.56 | 34.22 ± 1.90 | 1.28 | *; P = 0.0385 |
| Lung Fe(II) | 74.00 ± 9.07 | 82.11 ± 9.96 | 1.11 | n.s. |
| Nasopharynx Fe(II) | 112.66 ± 10.13 | 182.45 ± 12.88 | 1.62 | **; P = 0.0037 |
| Blood Cu(II) | 11.01 ± 0.13 | 38.64 ± 1.72 | 3.51 | ****; P < 0.0001 |
| Brain Cu(II) | 10.24 ± 0.45 | 12.42 ± 1.05 | 1.21 | n.s. |
| Lung Cu(II) | 6.30 ± 0.29 | 10.04 ± 1.29 | 1.59 | n.s. |
| Nasopharynx Cu(II) | 6.33 ± 0.91 | 5.40 ± 0.54 | 0.85 | n.s. |
| Blood Zn(II) | 15.60 ± 0.72 | 641.35 ± 173.28 | 41.11 | *; P = 0.026 |
| Brain Zn(II) | 41.96 ± 0.61 | 124.31 ± 15.25 | 2.96 | **, P = 0.0024 |
| Lung Zn(II) | 44.17 ± 2.59 | 103.36 ± 14.20 | 2.34 | *, P = 0.013 |
| Nasopharynx Zn(II) | 40.78 ± 3.94 | 214.45 ± 43.64 | 5.26 | *; P = 0.0162 |

^a^ P-values of <0.05, <0.005 and <0.0001 are denoted by *, ** or ****, respectively while comparisons that were not significant are denoted by n.s.
