# Supplementary material for: A Molecular Mechanism for Bacterial Susceptibility to Zinc
Source: PLoS Pathog. 2011 Nov 3;7(11):e1002357. doi: 10.1371/journal.ppat.1002357 (PMC3207923; doi:10.1371/journal.ppat.1002357)
Supplement: Table S5 — Oligonucleotide primers used in this study. (DOC) [file ppat.1002357.s008.doc]

**Table S5**. Oligonucleotide primers used in this study.

| **Primer** | **Sequence (5’→3’)** | **Comment** |
| --- | --- | --- |
| AD16 (F) | ATTGTAACCAGCCAAGGAGCATTC | For E205→Q205 mutation |
| AB36 (R) | AGCAAAAGGTGGATCCATTGAAAC | For E205→Q205 and D280→N280 mutation; *Bam*HI site underlined |
| AD6 (F) | CTTCTGGATCCAAACTAAAAGTTGTTGCTAC | For *Bam*HI/*Kpn*I cloning into pQE30, *Bam*HI site underlined |
| AD17 (R) | TGCTCCTTGGCTGGTTACAATGAG | For E205→Q205 mutation |
| AD18 (F) | CAAATCTTTACTAACTCTATCGCA | For D280→N280 mutation |
| AD19 (R) | GATAGAGTTAGTAAAGATTTGTGC | For D280→N280 mutation |
| AB24 (R) | CGTTTTGGTACCGCTTATTTTGCCAATCC | For *Bam*HI/*Kpn*I cloning into pQE30; *Kpn*I site underlined |
| PsaA (F) | GGTACATTACTCGTTCTCTTTCTTTCT | For real-time RT-PCR |
| PsaA (R) | GTGTGGGTCTTCTTTTCCTTTTTC | For real-time RT-PCR |
| 16S rRNA (F) | GGTGAGTAACGCGTAGGTAA | For real-time RT-PCR |
| 16S rRNA (R) | ACGATCCGAAAACCTTCTTC | For real-time RT-PCR |
