# Supplementary material for: A Molecular Mechanism for Bacterial Susceptibility to Zinc
Source: PLoS Pathog. 2011 Nov 3;7(11):e1002357. doi: 10.1371/journal.ppat.1002357 (PMC3207923; doi:10.1371/journal.ppat.1002357)
Supplement: Table S6 — PsaA-Mn(II) structure data collection and refinement statistics. (DOC) [file ppat.1002357.s009.doc]

**Table S6. Mn(II)-PsaA structure data collection and refinement statistics**

| **Data collection statistics** |  |
| --- | --- |
| Wavelength (Å) | 1.89445 |
| Data collection temperature (K) | 100 |
| Space group | *P*2_1_ |
| Unit cell parameters: a, b, c (Å); α, β, γ (°) | 68.7, 108.0, 78.3; 90.0, 95.8, 90.0 |
| Resolution range (outer shell in brackets; Å) | 19.8 – 2.74 (2.89 – 2.74) |
| Unique reflections | 28,404 |
| Total observations | 96,596 |
| <I / σ(I)> all (outer shell) | 9.3 (1.7) |
| R_merge_ all (outer shell) (%)^a^ | 9.6 (56.7) |
| Completeness all (outer shell) (%) | 99.2 (73.1) |
| Multiplicity | 3.4 |
| Wilson B-factor (Å^2^) | 75.1 |
| **Refinement statistics** |  |
| Amino acid residues (chains A, B, C, D) | 32-309, 32-309, 32-309, 32-309 |
| Non-hydrogen protein atoms in the model | 9,025 |
| Metal ions in the model | 4 × Mn(II) |
| Water molecules in the model | 154 |
| Solvent content (%) | 45.1 |
| Bond length deviation from ideal values (Å) | 0.016 |
| Bond angle deviation from ideal values (°) | 1.629 |
| Dihedral angle deviation from ideal values (°) | 18.8 |
| Average B-factor (Å^2^) | 37.7 |
| Ramachandran favored/outliers (%) | 93.8/0.4 |
| Reflections used (working/free) | 27,892/1,487 |
| R_work_/R_free_ (%)^b^ | 21.5/24.6 |
| MolProbity score (43^rd^ percentile) | 2.96 |
| Cruickshank’s DPI for coordinate error (Å)^c^ | 0.36 |

^a^ R_merge_ = ∑*_hkl_*(∑*_i_*(|I *_hkl,i_*-<I *_hkl_* >|))/∑*_hkl,i_* <I *_hkl_*>, where I *_hkl,i_* is the intensity of an individual measurement of the reflection with Miller indices h, k and l, and <I_hkl_> is the mean intensity of that reflection. Calculated for I > -3σ(I).

^b^ R_work_ = ∑*_hkl_*(||Fobs*_hkl_*|-|Fcalc*_hkl_*||)/|Fobs*_hkl_*|, where |Fobs*_hkl_*| and |Fcalc*_hkl_*| are the observed and calculated structure factor amplitudes.

R_free_ = R_free_ is equivalent to R_work_ but calculated using the reflections (5 %) omitted from the refinement process.

^c^ Cruickshank’s DPI is calculated using the R-factor, the number of reflections and parameters with the data completeness taken into account.
