# Supplementary material for: A Molecular Mechanism for Bacterial Susceptibility to Zinc
Source: PLoS Pathog. 2011 Nov 3;7(11):e1002357. doi: 10.1371/journal.ppat.1002357 (PMC3207923; doi:10.1371/journal.ppat.1002357)
Supplement: Text S1 — Isothermal calorimetric analysis of PsaA. Interpretation of the ITC analysis of PsaA presented in Fig. 1A and B. (DOC) [file ppat.1002357.s010.doc]

**Text S1**

**PsaA is the high affinity Mn solute binding protein**

The PsaA-Mn(II) binding isotherm (Fig. 1A) showed a very strong interaction. The Zn(II) binding isotherm (Fig. 1B) with *apo*-PsaA also showed a strong interaction, but had significant complexity as the ratios approached 1:1 presumably due to the polyhistidine tag on the recombinant protein.

Further investigation of the metal binding capacity of PsaA was conducted using saturating (100-fold excess) concentrations of Mn(II) or Zn(II). Analysis by inductively coupled plasma mass spectroscopy (ICPMS) found that the purified Mn(II) loaded protein had 1.24 mol Mn(II)/mol PsaA, while Zn(II) loaded PsaA contained 5.40 mol Zn(II)/mol of protein (Table S1). The higher Zn(II) loading under saturating conditions supports the ITC observations for the presence of lower affinity sites Zn(II) binding sites in PsaA, which are, in all likelihood, associated with the polyhistidine tag. Notably the mutant isoforms of PsaA, which had the metal coordinating O-ligands from Glu205 or Asp280 mutated to their neutral polar analogues Gln205 or Asn280, had an impaired capacity for metal binding (Table S1).
