# Supplementary material for: A Molecular Mechanism for Bacterial Susceptibility to Zinc
Source: PLoS Pathog. 2011 Nov 3;7(11):e1002357. doi: 10.1371/journal.ppat.1002357 (PMC3207923; doi:10.1371/journal.ppat.1002357)
Supplement: Text S2 — Crystallographic analysis of PsaA-Mn(II). Interpretation of the PsaA-Mn(II) crystal structure presented in Figs. 1C, D, and S1. (DOC) [file ppat.1002357.s011.doc]

**Text S2**

**Structural analysis of Mn(II) and Zn(II) binding**

Well-defined and continuous electron density was observed for all the metal-liganding residues. Several pieces of evidence support our interpretation that the structure contains Mn(II). First, the anomalous difference Fourier map computed based on diffraction data set collected at the manganese K-edge peak wavelength of 1.844 Å shows a clear peak at the 4σ level at the corresponding metal ion positions in all four molecules in the asymmetric unit. The f’’ values for Mn and Zn atoms at this wavelengths are 4 and 1, respectively, and the size of the anomalous density peak is strongly indicative of the anomalous scattering resulting from the presence of manganese. Second, the X-ray fluorescence spectrum obtained at the synchrotron confirmed the presence of manganese in the crystal. Third, the crystallographic refinement statistics are also consistent with the metal being Mn(II); the average atomic displacement parameter (*B*-factor) for the metal when modelled as Mn(II) is 30.7 Å^2^, which compares well with the 24.7 Å^2^ average atomic displacement parameter for the coordinating residues. By contrast, when the metal was modelled as Zn(II), the average atomic displacement parameter of the metal ion increases to 56.5 Å^2^, 33.7 Å^2^ higher than that of the coordinating residues.
