# Supplementary material for: A Molecular Mechanism for Bacterial Susceptibility to Zinc
Source: PLoS Pathog. 2011 Nov 3;7(11):e1002357. doi: 10.1371/journal.ppat.1002357 (PMC3207923; doi:10.1371/journal.ppat.1002357)
Supplement: Text S3 — Supporting Methods and References. (DOC) [file ppat.1002357.s012.doc]

**Text S3**

**Thermal Shift Assay**

The thermal shift assay was based on the Thermfluor assay conducted using 10 μM PsaA in 100 mM MOPS, pH 7.2, 150 mM NaCl, 5× SYPRO Orange (Invitrogen) in the presence of 10 μM to 1000 μM metal, using a Roche LC480 Real-Time Cycler. The samples were pre-incubated for 10 min with the indicated metal ion concentration and then subjected to thermal unfolding from 25°C to 97°C at a heating rate of 1°C per min. Fluorescence data were also collected for buffer only, PsaA without metal ions, and for each of the metal ions without protein. The fluorescence data were collected by excitation at 470 nm and emission at 570 nm. After subtraction of the background fluorescence from the buffer, the first derivative of the fluorescence data was determined and analyzed using Graphpad Prism to determine the inflection point of the melting transition (T*_m_*).

**Crystallographic data refinement**

The diffraction data were collected on a single crystal and the reflections were indexed and integrated using *XDS* and scaled with *SCALA*. Mn-containing crystals exhibited the space group P2_1_, different to that of crystals of the Zn-bound form reported previously . The unit cell was estimated to have four PsaA monomers in the asymmetric unit and phases were obtained by rotation and translation search using the molecular replacement method as implemented in *PHASER*  with the structure of the Zn-bound PsaA (pdb code: 1PSZ) as the search mode . The model was subjected to six cycles of automated building and refinement in *PHENIX*. The models were manually inspected and rebuilt where necessary in *COOT*  followed by maximum likelihood restrained positional refinement in *REFMAC*. Strict main chain and medium side-chain non-crystallographic symmetry (NCS) restraints for all four chains were applied throughout refinement. Translation, libration and screw (TLS) restraints were included in the refinement protocol. Stereochemistry of the final models was validated using *MOLPROBITY*.

**PMN Killing Assay**

Dextran-sedimented PMNs were purified on a Ficoll-Hypaque gradient. PMNs were resuspended at 2.4 × 10^6^ cells.ml^−1^ in RPMI + 10% heat inactivated fetal bovine serum (FBS) and kept on ice until use. PMN preparations routinely contained >95% PMNs, assessed morphologically by phase-contrast microscopy, and viability, monitored by trypan blue exclusion, was >99%. The adherent, PMN assay with *S. pneumoniae* was performed in triplicate with replicate PMN monolayers. Bacterial cells were grown to an *A*_600_ = 0.3, washed 3 times with PBS + 2.5 mM EDTA and then 3 times with PBS. A 1:1 ratio of PMNs to *S. pneumoniae* was prepared and the cells brought into contact by centrifugation at 600 x *g* for 4 minutes and the mixture aspirated. Cells were incubated for 90 minutes with adherent neutrophils in 1 ml of RPMI + FBS and then washed 3 times with RPMI + FBS to remove unbound bacteria. The mixture was treated for 10 minutes with 1% saponin after which serial dilutions were plated on blood agar plates. Survival was calculated as a percentage of bacterial cells at 90 minutes compared to 0 minutes.

Supporting References

1. Cummings MD, Farnum MA, Nelen MI (2006) Universal screening methods and applications of ThermoFluor. J Biomol Screen 11: 854-863.

2. Kabsch W (1993) Automatic processing of rotation diffraction data from crystals of initially unknown symmetry and cell constants. J Appl Crystallogr 26: 5.

3. Evans P (2006) Scaling and assessment of data quality. Acta Crystallogr D Biol Crystallogr 62: 72-82.

4. Lawrence MC, Pilling PA, Epa VC, Berry AM, Ogunniyi AD, et al. (1998) The crystal structure of pneumococcal surface antigen PsaA reveals a metal-binding site and a novel structure for a putative ABC-type binding protein. Structure 6: 1553-1561.

5. McCoy AJ, Grosse-Kunstleve RW, Adams PD, Winn MD, Storoni LC, et al. (2007) Phaser crystallographic software. J Appl Crystallogr 40: 658-674.

6. Terwilliger TC, Grosse-Kunstleve RW, Afonine PV, Moriarty NW, Zwart PH, et al. (2008) Iterative model building, structure refinement and density modification with the PHENIX AutoBuild wizard. Acta Crystallogr D Biol Crystallogr 64: 61-69.

7. Emsley P, Cowtan K (2004) Coot: model-building tools for molecular graphics. Acta Crystallogr D Biol Crystallogr 60: 2126-2132.

8. Murshudov GN, Vagin AA, Dodson EJ (1997) Refinement of macromolecular structures by the maximum-likelihood method. Acta Crystallogr D Biol Crystallogr 53: 240-255.

9. Painter J, Merritt EA (2006) Optimal description of a protein structure in terms of multiple groups undergoing TLS motion. Acta Crystallogr D Biol Crystallogr 62: 439-450.

10. Chen VB, Arendall WB, 3rd, Headd JJ, Keedy DA, Immormino RM, et al. (2010) MolProbity: all-atom structure validation for macromolecular crystallography. Acta Crystallogr D Biol Crystallogr 66: 12-21.
